# A clinical-radiomics combined model based on carotid atherosclerotic plaque for prediction of ischemic stroke

**DOI:** 10.3389/fneur.2024.1343423

**Published:** 2024-03-13

**Authors:** Na Han, Wanjun Hu, Yurong Ma, Yu Zheng, Songhong Yue, Laiyang Ma, Jie Li, Jing Zhang

**Affiliations:** ^1^Department of Magnetic Resonance, Lanzhou University Second Hospital, Lanzhou, China; ^2^Gansu Province Clinical Research Center for Functional and Molecular Imaging, Lanzhou, China; ^3^Second Clinical School, Lanzhou University, Lanzhou, China

**Keywords:** stroke, carotid, atherosclerotic plaques, radiomics, magnetic resonance imaging, high-resolution vessel wall

## Abstract

**Objectives:**

To accurately predict the risk of ischemic stroke, we established a radiomics model of carotid atherosclerotic plaque-based high-resolution vessel wall magnetic resonance imaging (HR-VWMRI) and combined it with clinical indicators.

**Materials and methods:**

In total, 127 patients were finally enrolled and randomly divided into training and test cohorts. HR-VWMRI three-dimensional T1-weighted imaging (T1WI) and contrast-enhanced T1WI (T1CE) were collected. A traditional model was built by recording and calculating radiographic features of the carotid plaques and patients’ clinical indicators. After extracting radiomics features from T1WI and T1CE images, the least absolute shrinkage and selection operator (LASSO) algorithm was used to select the optimal features and construct the radiomics_T1WI model and the radiomics_T1CE model. The traditional and radiomics features were used to build combined models. The performance of all the models predicting ischemic stroke was evaluated in the training and test cohorts, respectively.

**Results:**

Body mass index (BMI) and intraplaque hemorrhage (IPH) were independently related to ischemic stroke and were used to build the traditional model, which achieved an area under the curve (AUC) of 0.79 versus 0.78 in the training and test cohorts, respectively. The AUC value of the radiomics_T1WI model is the lowest in the training and test cohorts, but the prediction performance is significantly improved when the model combines IPH and BMI. The AUC value of the combined_T1WI model was 0.78 and 0.81 in the training and test cohorts, respectively. In addition, in the training and test cohorts, the radiomics_T1CE model based on HR-VWMRI combined clinical characteristics, which is the combined_T1CE model, had the highest AUC value of 0.84 and 0.82, respectively.

**Conclusion:**

Compared with other models, the radiomics_T1CE model based on HR-VWMRI combined clinical characteristics, which is a combined_T1CE model, can accurately predict the risk of ischemic stroke.

## Introduction

In 2015, the global mortality rate of ischemic cerebrovascular disease rose to second place among all causes of death. At the beginning of 2017, the China Stroke Epidemiology Survey team reported on the Circulation that the prevalence rate of stroke in China reached 1148.3/100000 after age standardization, of which ischemic stroke accounted for more than 70%, which has become a major disease seriously threatening the health of Chinese people (1–3). Atherosclerosis, a major cause of ischemic stroke, is a chronic progressive disease characterized by the atherosclerotic plaque formation. Embolism caused by carotid atherosclerotic plaque shedding has been recognized as accounting for approximately 18 to 25% of all strokes (4). In 2018, the American Society of Neuroradiology (ASNR) Vessel Wall Imaging Study Group published guidelines highlighting that the risk and severity of stroke associated with carotid plaques are not only related to the extent of luminal stenosis, but also related to plaque characteristics (5). An increasing amount of evidence indicates that vulnerable plaques are highly likely to lead to ischemic stroke and thrombotic complications, independent of the extent of luminal stenosis (6, 7). High-resolution vessel wall magnetic resonance imaging (HR-VWMRI) can not only assess luminal stenosis, but also characterize plaque morphology and different atherosclerotic components and identify vulnerable plaques (8). Plaque vulnerability imaging features include intraplaque hemorrhage (IPH), lipid-rich necrotic cores (LRNC) and thin fibrous caps, plaque inflammation, intraplaque neovascularization, plaque surface ulceration, and positive vascular remodeling (9). Randomized clinical trials have shown that HR-VWMRI is the most suitable and cost-effective imaging technique for characterizing plaque vulnerability characteristics. However, there are still drawbacks. First, HR-VWMRI has a longer scanning time, and image quality is sensitive to motion. Second, identifying vulnerable plaques based on HR-VWMRI is qualitative and subjective, and the results are greatly influenced by the personal factors of the researchers. Third, there is currently a lack of multicenter, large-scale, high-quality research on the relationship between vulnerable plaque characteristics and the risk of ischemic stroke. However, the relationship between carotid atherosclerotic plaque and ischemic stroke should go beyond the assessment of the primary imaging characteristics of plaque or the degree of vascular stenosis and adopt a new model in which carotid plaque imaging combined with artificial intelligence.

As an emerging multidisciplinary research field, radiomics integrates digital imaging information, statistics, artificial intelligence, machine learning, and deep learning methods to convert medical images into high-throughput quantitative features for research. Radiomics has been applied to disease diagnosis, tumor staging or grading, gene prediction, therapeutic effect evaluation, and prognosis judgment and plays an important role in assisting clinical decision-making. At present, radiomics studies on atherosclerotic plaques focus on identifying vulnerable plaques (10–12). The preliminary radiomics study of atherosclerotic plaque is based on texture analysis of CT or US images (13, 14). Compared with CT and US, VW-HRMRI has advantages in high soft tissue contrast and multisequence imaging, which can provide more valuable information. Shi et al. (11) conducted a radiomic study that was based on VW-HRMRI to distinguish stable and vulnerable basilar artery plaques. Subsequently, Shi et al. (15) performed a histogram texture analysis that was based on VW-HRMRI to extract the first-order texture features of atherosclerotic plaques in the middle cerebral artery and basilar artery, and they explored the differences in histogram features between stable and vulnerable plaques. Zhang et al. (16) established a high-risk carotid plaque model based on MRI radiomic features and evaluated its performance in distinguishing stable and vulnerable carotid plaques relative to the model based on traditional MRI features. It can be seen that the radiomic model showed better performance than the traditional model. These results indicate that compared with the traditional subjective qualitative and quantitative imaging characteristics, the radiomic method enables finding more differential features, showing its higher value in determining plaque vulnerability. This study established a radiomics model of carotid plaque based on HR-VWMRI and combined it with clinical indicators to accurately predict the risk of ischemic stroke.

## Materials and methods

### Study population

A total of 182 patients with a carotid plaque were consecutively recruited in this study between January 2020 and March 2022 in our hospital. All of them underwent HR-VW MRI 3D T1WI, T1CE, and head MRI (T1WI, T2WI, DWI, FLAIR, and 3D TOF-MRA). The study was approved by the ethics review board of our hospital (scientific research project ethics approval number: 2022A-695).

**Figure 1:**
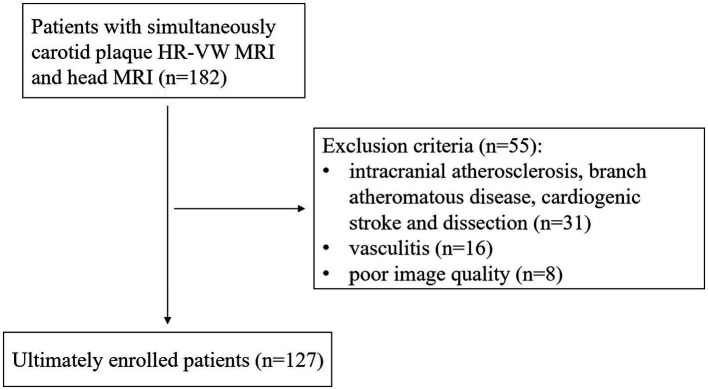
Study inclusion and exclusion flowchart.

The exclusion criteria were as follows: (a) patients with cardiogenic stroke; (b) intracranial atherosclerosis, branch atheromatous disease, other causes (such as dissection, vasculitis, and vascular malformations), and causes remain unknown on ipsilateral infarction; (c) primary intracranial diseases; and (d) poor image quality. In total, 55 patients were excluded because of intracranial atherosclerosis, branch atheromatous disease, cardiogenic stroke and dissection on ipsilateral infarction (*n* = 31), vasculitis (*n* = 16), and poor image quality (*n* = 8), and 127 patients were finally enrolled in our study (flowchart in Figure 1). All patients were divided into stroke and stroke-free groups according to whether acute/subacute stroke (carotid territory) was shown on head MRI. We recorded the demographic and clinical characteristics of all participants including gender, age, body mass index (BMI), hypertension, hyperglycemia, hyperlipidemia, hyperuricemia, and hyperhomocysteinemia. Eligible patients were randomly divided into a training cohort (*n* = 75) and a test cohort (*n* = 52) with a ratio of approximately 3: 2.

### MRI acquisition

The HR-VWMRI and head MRI were performed on a 3.0T MR scanner with a 20-channel head-and-neck phased array coil. Carotid MRI sequences include 3D TOF-MRA, 3D T1WI, T2WI, and 3D T1CE. Head MRI sequences include T1WI, T2WI, DWI, FLAIR, and 3D TOF-MRA. Detailed scanning parameters are listed in Table 1. The total scanning time was approximately 29 min.

**Table 1:** Detailed scanning parameters for the MRI protocol.

Sequence	Carotid plaque MRI				Head MRI				
	TOF	T1WI	T2WI	T1CE	T1WI	T2WI	DWI	FLAIR	TOF
FOV (mm)	220 × 128	240 × 240	160 × 160	240 × 240	230 × 230	230 × 230	230 × 230	230 × 230	230 × 230
Matrix	292 × 961	344 × 344	268 × 255	344 × 344	296 × 167	288 × 288	152 × 122	256/173	400 × 242
Slice thickness (mm)	1.2	0.8	3	0.8	5.5	5.5	5.5	5.5	1.2
Resolution (mm^2^)	0.6 × 1.0	0.7 × 0.7	0.6 × 0.6	0.7 × 0.7	1.0 × 1.2	0.6 × 0.6	1.5 × 1.9	0.9 × 0.9	0.6 × 1.0
TR/TE (ms)	15/3.5	500/26	3500/60	500/26	2000/20	4000/97	2887/97	7500/110	22/3.5
NEX	1	1	2	1	1	1	1	1	1
Bandwidth (Hz/pixel)	216	637	273	637	159	439	1448	514	109
Flip angle (°)	18	75	90	75	90	90	90	90	18
Acquisition time	3min10s	6min46s	4min05s	6min46s	1min30s	2min24s	30s	1min21s	2min47s

TOF, time of flight; DWI, diffusion-weighted imaging; FLAIR, fluid-attenuated inversion recovery; FOV, field of view; TR, repetition time; TE, echo time; NEX, number of excitations.

### Image analysis and segmentation

Independently identified and measured radiological features of the carotid plaques including IPH, LRNC, disrupted surface, enhancement, remodeling pattern, maximum wall area (Max WA), normalized wall index (NWI), and degree of stenosis by two senior radiologists (N.H. and YR.M.) with 8 or more years of experience in plaque imaging. If there are differences, the two radiologists reached a consensus after an additional reading session. Qualitative analysis of carotid plaques was performed on Picture Archiving and Communication Systems (PACS). For quantitative analysis of carotid plaques, VesselMass software (MEDIS, Version: 2014-EXP), a semi-automatic image analysis tool, was used. The slice with the largest plaque area was chosen, and the outer wall boundaries and inner lumen were manually outlined for measuring and calculating the total vessel area (TVA), the minimum luminal area (Min LA), the maximum wall area (Max WA), the normalized wall index (NWI), the remodeling index (RI), and the degree of stenosis. The relevant calculation formulas were as follows: (1) Max WA = TVA-Min LA (17); (2) NWI = Max WA/TVA (18, 19); (3) RI = vessel area at the point of maximum stenosis/reference vessel area at the distal portion (7); (4) Stenosis = (normal diameter at the distal portion-narrowest diameter at the stenosis)/normal diameter at the distal portion (20). If the RI is greater than 1, it is defined as positive remodeling; otherwise, it is defined as negative remodeling.

**Figure 2:**
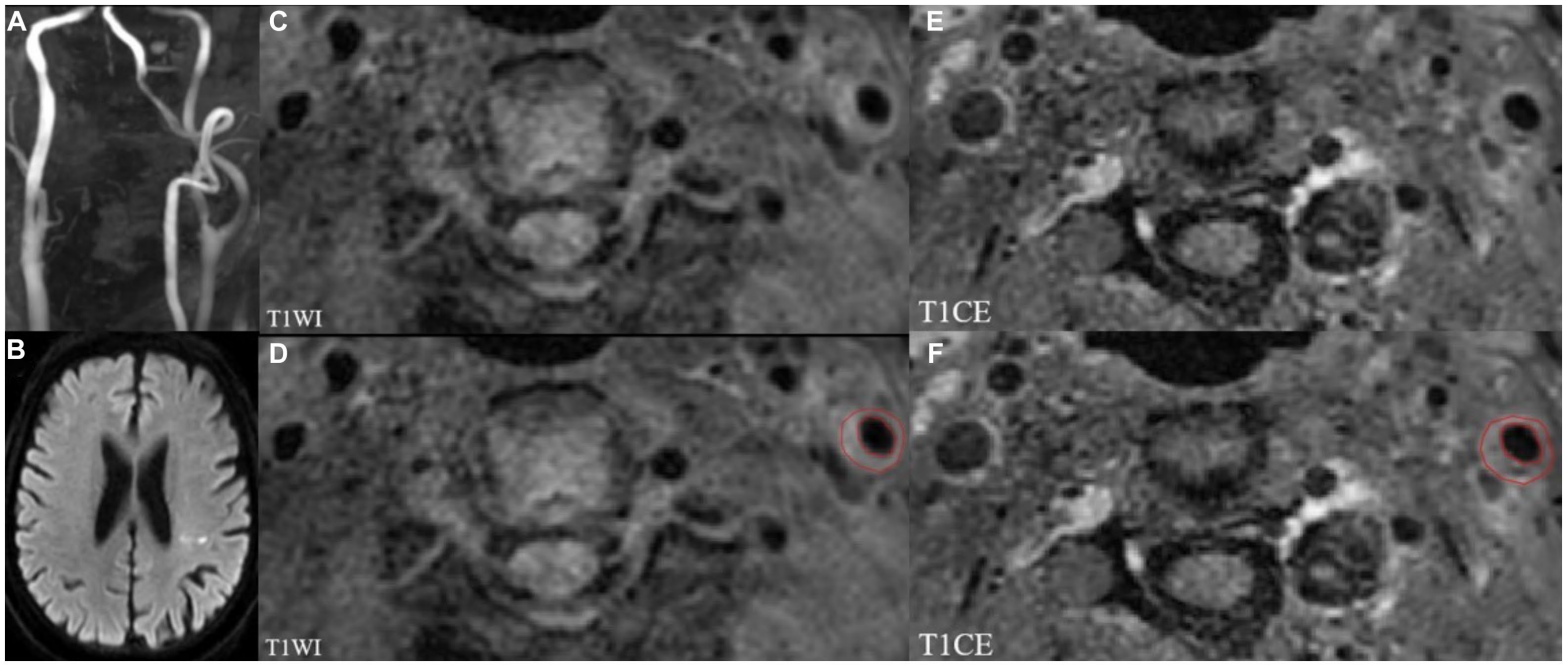
MRI images showing left internal carotid atherosclerotic plaque in a stroke patient. TOF-MRA **(A)** demonstrates mild stenosis, and DWI **(B)** shows the acute infarcts which are scattered and patchy in distribution within the left lateral ventricle posterior horn around. Left internal carotid atherosclerotic plaque on T1WI and T1CE images shown in panels **(C,E)**. The regions of interest (ROIs) of the carotid atherosclerotic plaque on T1WI and T1CE images **(D,F)**.

The open-source software ITK-SNAP (version 3.8.0)^1^ was used to plaque segmentation for radiomics analysis. Volumes of interest (VOIs) were manually drawn layer by layer on different sequences including HR-VWMRI 3D T1WI and T1CE by the above two senior radiologists along the margin of the plaques, respectively. The images of the sample patient are shown in Figure 2.

Randomly selected 40 cases to evaluated interobserver and intraobserver reproducibility using intraclass correlation coefficients (ICC). Radiologist H manually outlined the VOIs twice within three months, ICCs>0.75 indicated good consistency of intraobserver. Radiologist M outlined the VOIs once, ICCs>0.75 indicated good consistency of interobserver. Radiologist H completed the remaining outline.

### Feature extraction, selection, and model development

HR-VWMRI 3D T1WI and 3D T1CE images were used for feature extraction by applying the Pyradiomics package of Python software.^2^ The extracted features include shape features, first-order statistics features, second-order statistics features, and higher order statistics features. In order to standardize and normalize the original features of different dimensions, the min–max standardization method is used to linearly transform the original feature dataset and map the values between 0 and 1. First, the features of *p* < 0.05 in each sequence were selected using Student’s *t*-test. Second, the LASSO algorithm was used for selecting optimal feature subsets by penalty function λ adjust and set the corresponding coefficient of features with weak correlation to 0. Selection of the tuning parameter (λ) in the LASSO model via 10-fold cross-validation based on minimum criteria. The final retained features with non-zero coefficients and using non-zero coefficient features construct radiomics model. The radiomics score (Rad-Score) calculation used a linear combination of select parameters weighted by the relevant LASSO coefficients.

**Figure 3:**
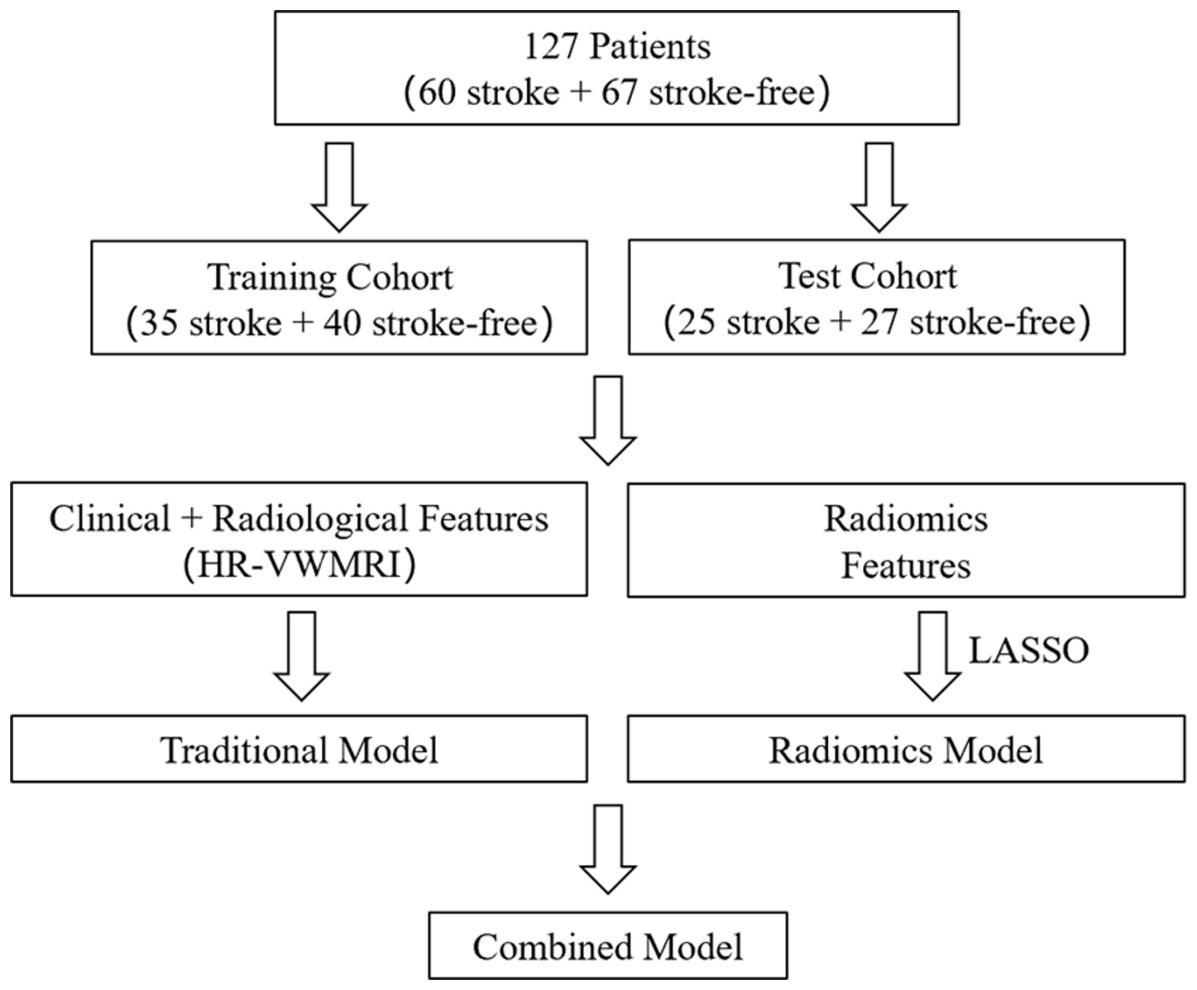
Flowchart for building the models predicting ischemic stroke.

We selected the clinical and radiological characteristics with *p* < 0.05 in univariate analysis for multivariate logistic regression analysis, by which the odds ratios (ORs) with 95% confidence intervals (CIs) were calculated. The variables with *p* < 0.05 in multivariate analysis were finally used to establish the traditional model. The traditional model and radiomics features were combined to establish the combined model, which were displayed as radiomics nomograms (flowchart in Figure 3). In the training process of all models, to avoid overfitting of models, a 10-fold cross-validation method was used, and then, the prediction performance of all models in the training and test cohorts was evaluated, respectively.

### Statistical analysis

All statistical analyses were performed using the IBM SPSS Statistics software (version 25.0) and Python software (see text footnote 2). The relationship between each variable and stroke status was evaluated by univariate analysis. The continuous variables adopted the Mann–Whitney *U*-test, and the categorical variables adopted the chi-squared test. Variables with *p* < 0.05 in univariate analysis were enrolled in multivariate logistic regression analysis. Receiver operating characteristic (ROC) analysis was used to determine the AUC values to evaluate the predictive performance of all models in both the training and test cohorts.

## Results

### Patient characteristics

In total, 127 patients with carotid plaque were enrolled in the final analysis, there were 60 stroke patients and 67 stroke-free patients. Table 2 lists the demographic and clinical characteristics of the enrolled patients.

**Table 2:** Demographic and clinical characteristics of the enrolled patients.

	*n*/total (%)	Stroke	Stroke-free	*p*-value^b^	Multivariate OR (95 %CI)^c^	*p*-value^c^	AUC
Sex	127	60	67	0.015^d^	0.168 (0.013, 2.205)	0.174	
Male	103	54	49				
Female	24	6	18				
Age^a^	60.55 ± 9.72	59.90 ± 10.90	61.20 ± 8.52	0.428^e^			
BMI^a^	24.81 ± 1.59	25.48 ± 1.68	24.22 ± 1.24	< 0.001^e^	2.564 (1.412, 4.656)	0.002	0.726
Hypertension	82	34	48	0.078^d^			
Hyperglycemia	43	22	21	0.527^d^			
Hyperlipidemia	36	15	21	0.428^d^			
Hyperuricemia	8	5	3	0.598^d^			
Hyperhomocysteinemia	75	39	36	0.197^d^			
IPH	45	36	9	< 0.001^d^	0.047 (0.005, 0.444)	0.008	0.733
LRNC	64	31	33	0.786^d^			
Disrupted surface	43	33	10	< 0.001^d^	0.527 (0.112, 2.490)	0.419	
Enhancement	41	25	16	0.017^d^	0.439 (0.082, 2.344)	0.335	
Remodeling pattern	127	60	67	0.509^d^			
Positive	80	36	44				
Negative	47	24	23				
Max WA (cm^2^)^a^	0.50 ± 0.23	0.56 ± 0.25	0.45 ± 0.18	0.017^e^	1.103 (0.047, 25.918)	0.951	
NWI^a^	0.75 ± 0.19	0.83 ± 0.15	0.67 ± 0.18	< 0.001^e^	0 (0, 119.173)	0.156	
Degree of stenosis (%)^a^	71.00 ± 23.00	81.00 ± 17.00	62.00 ± 23.00	< 0.001^e^	1.155 (0.994, 1.341)	0.060	

AUC, area under the curve; CI, confidence intervals; BMI, body mass index; IPH, intraplaque hemorrhage; LRNC, lipid-rich necrotic core; Max WA, maximum wall area; NWI, normalized wall index; OR, odds ratio.

^a^Continuous variables shown with mean ± standard deviation (SD), others are categorical variables; ^b^univariate analysis; ^c^results from multivariate logistic analysis; ^d^chi-squared test; ^e^Mann-Whitney U-test.

### Traditional model

**Figure 4:**
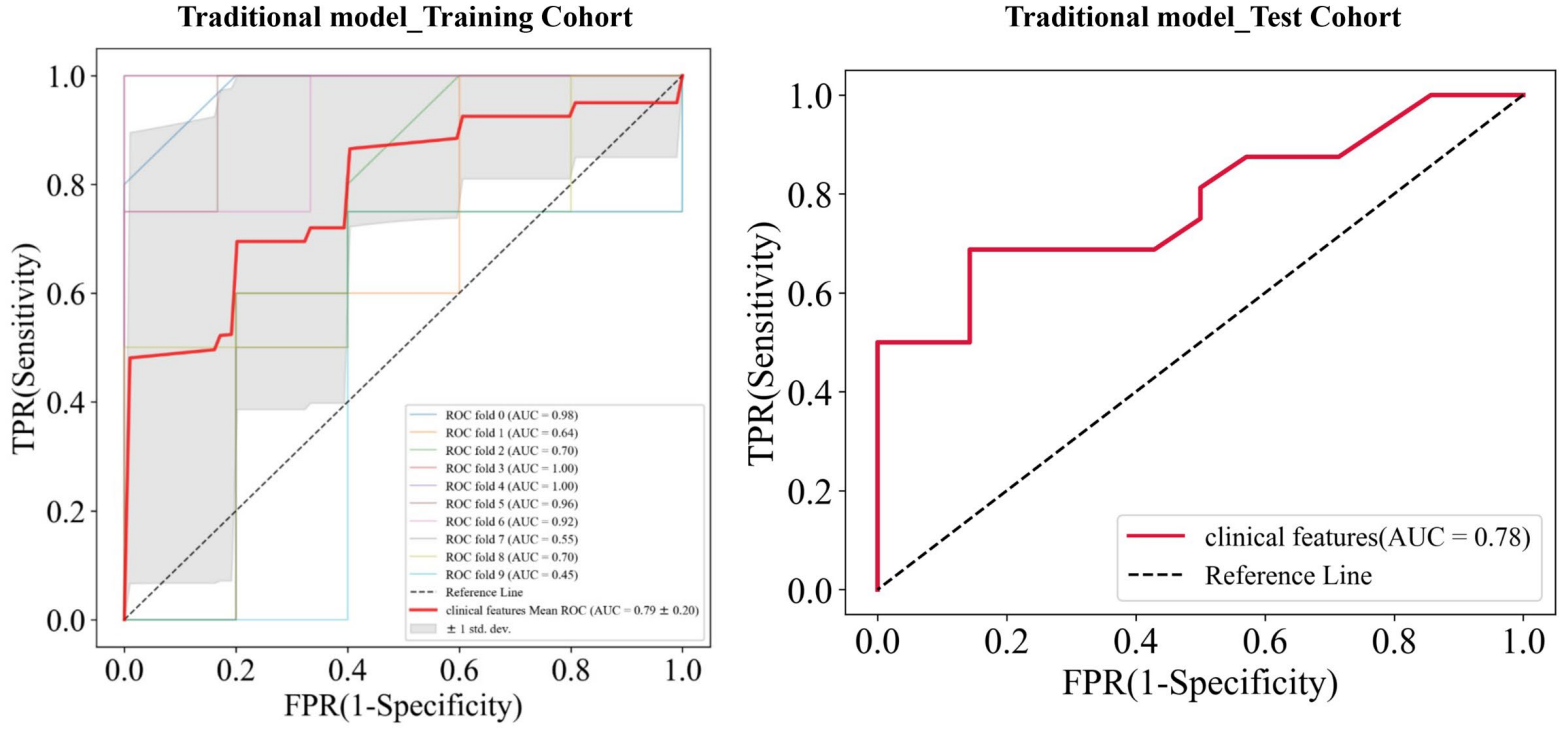
Receiver operating characteristic (ROC) curves of the traditional model in the training and test cohorts, respectively.

Univariate analysis showed that gender, BMI, IPH, disrupted surface, enhancement, Max WA, NWI, and degree of stenosis were significantly associated with stroke (all *p* < 0.05, Table 2). Multivariate logistic regression analysis indicated the BMI (OR = 2.564; 95% CI, 1.412–4.656) and IPH (OR = 0.047; 95% CI, 0.005–0.444) were independent predictors of ischemic stroke and were used to establish the traditional model. When combining BMI and IPH, the AUC values were 0.79 and 0.78 in the training and test cohorts, respectively (Figure 4).

### Radiomics model

**Figure 5:**
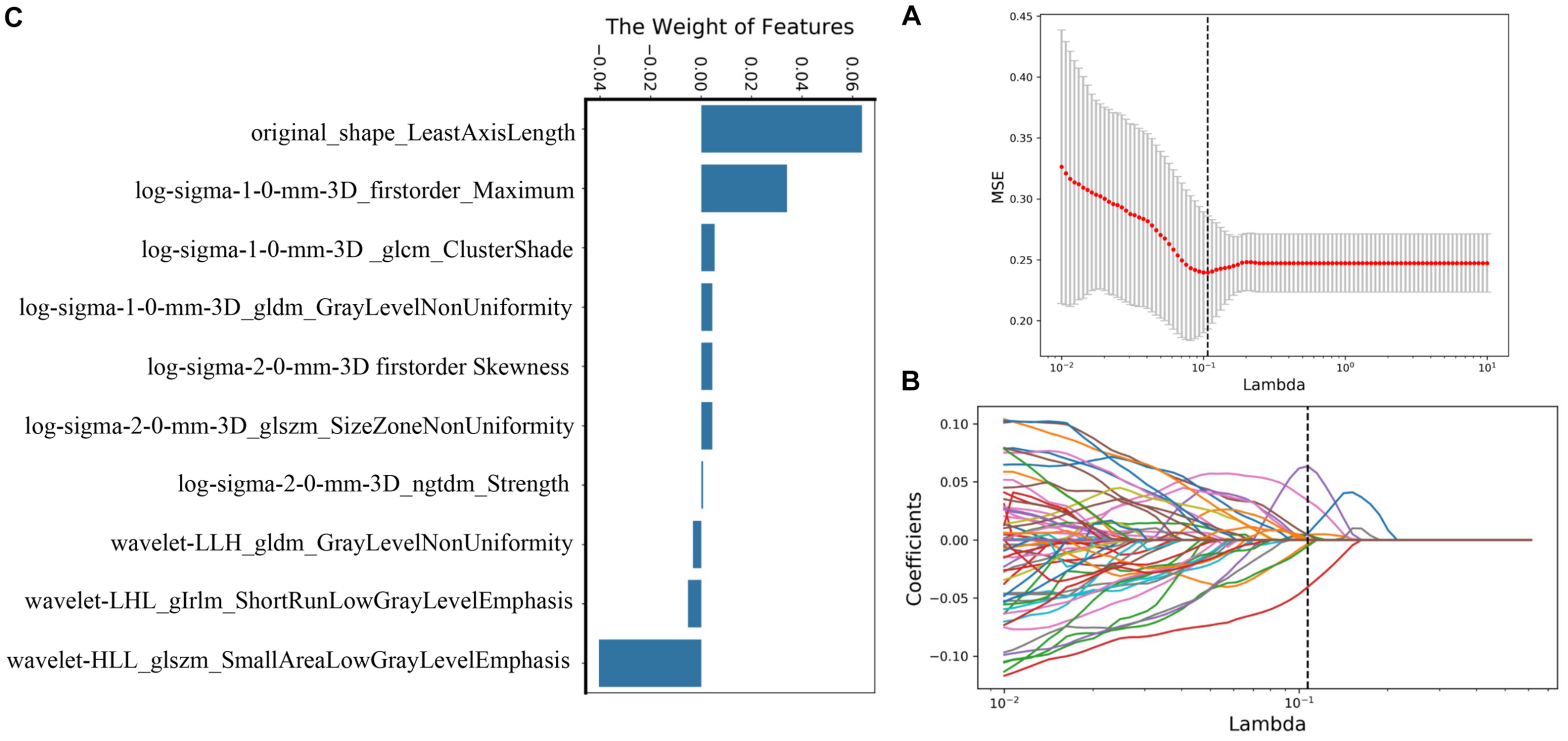
Selection of radiomics features using LASSO logistic regression based on T1WI images. **(A)** Selection of the tuning parameter (λ) in the LASSO model via 10-fold cross-validation based on minimum criteria. **(B)** The coefficients have been plotted versus (λ). **(C)** The final retained features with non-zero coefficients.

**Figure 6:**
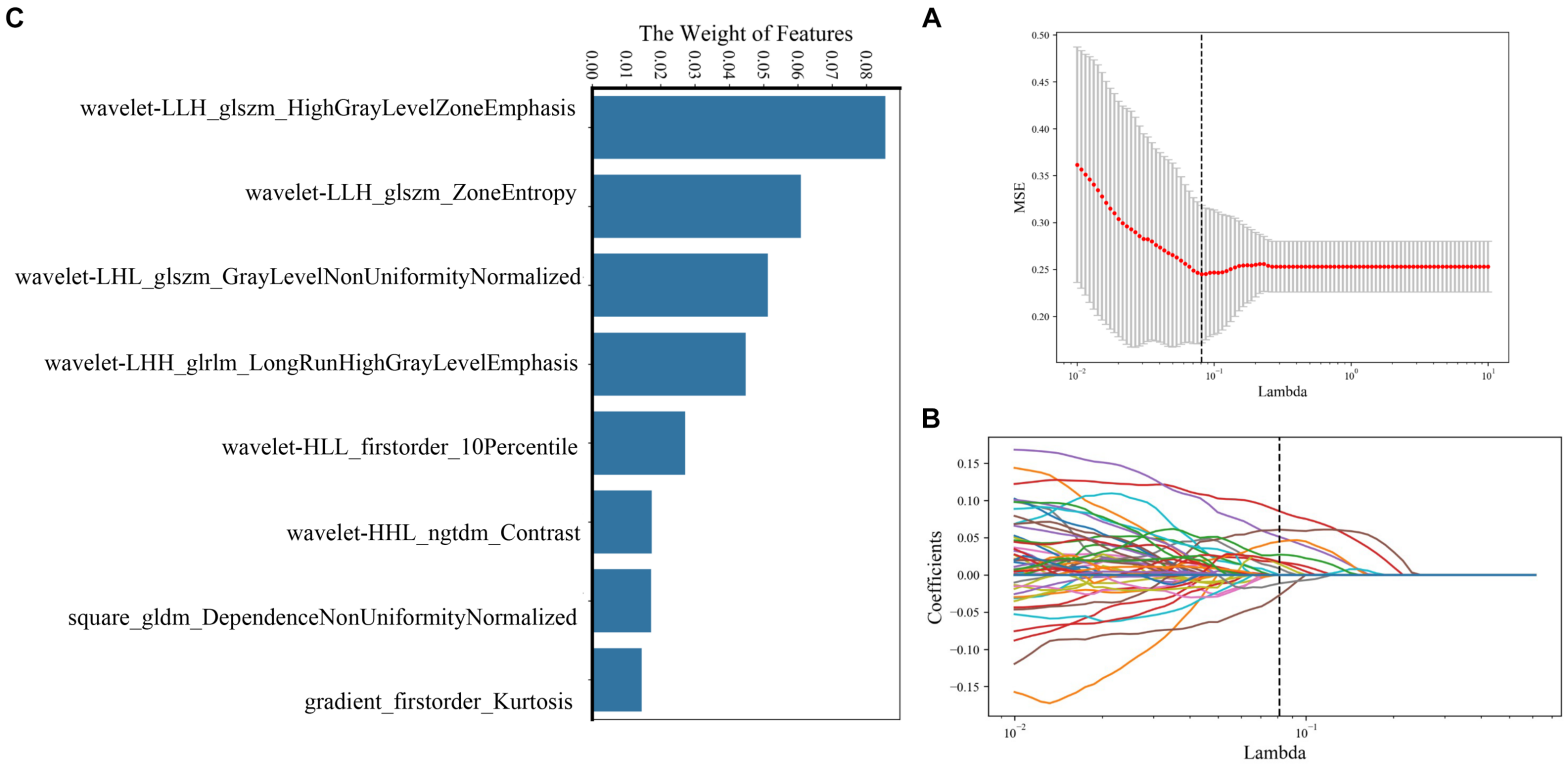
Selection of radiomics features using LASSO logistic regression based on T1CE images. **(A)** Selection of the tuning parameter (λ) in the LASSO model via 10-fold cross-validation based on minimum criteria. **(B)** The coefficients have been plotted versus (λ). **(C)** The final retained features with non-zero coefficients.

A total of 239 and 191 features were extracted from the whole plaque region based on T1WI and T1CE sequences, respectively. After the LASSO algorithm was applied, 10 and 8 features were finally retained, which were used to establish the radiomics_T1WI model and the radiomics_T1CE model, respectively. The radiomics_T1WI model includes one feature of shape, two feature of first-order statistics, and seven features of texture (one gray-level co-occurrence matrix (GLCM) features, two gray-level dependence matrix (GLDM) features, two gray-level size-zone matrix (GLSZM) features, one neighborhood gray-tone difference matrix (NGTDM) features, and one gray-level run-length matrix (GLRLM) features). The radiomics_T1CE model includes two features of first-order statistics and six features of texture (three gray-level size-zone matrix (GLSZM) features, one gray-level run-length matrix (GLRLM) features, one neighborhood gray-tone difference matrix (NGTDM) features, and one gray-level dependence matrix (GLDM) features). The screening process and final screening characteristics are shown in Figures 5, 6.

**Figure 7:**
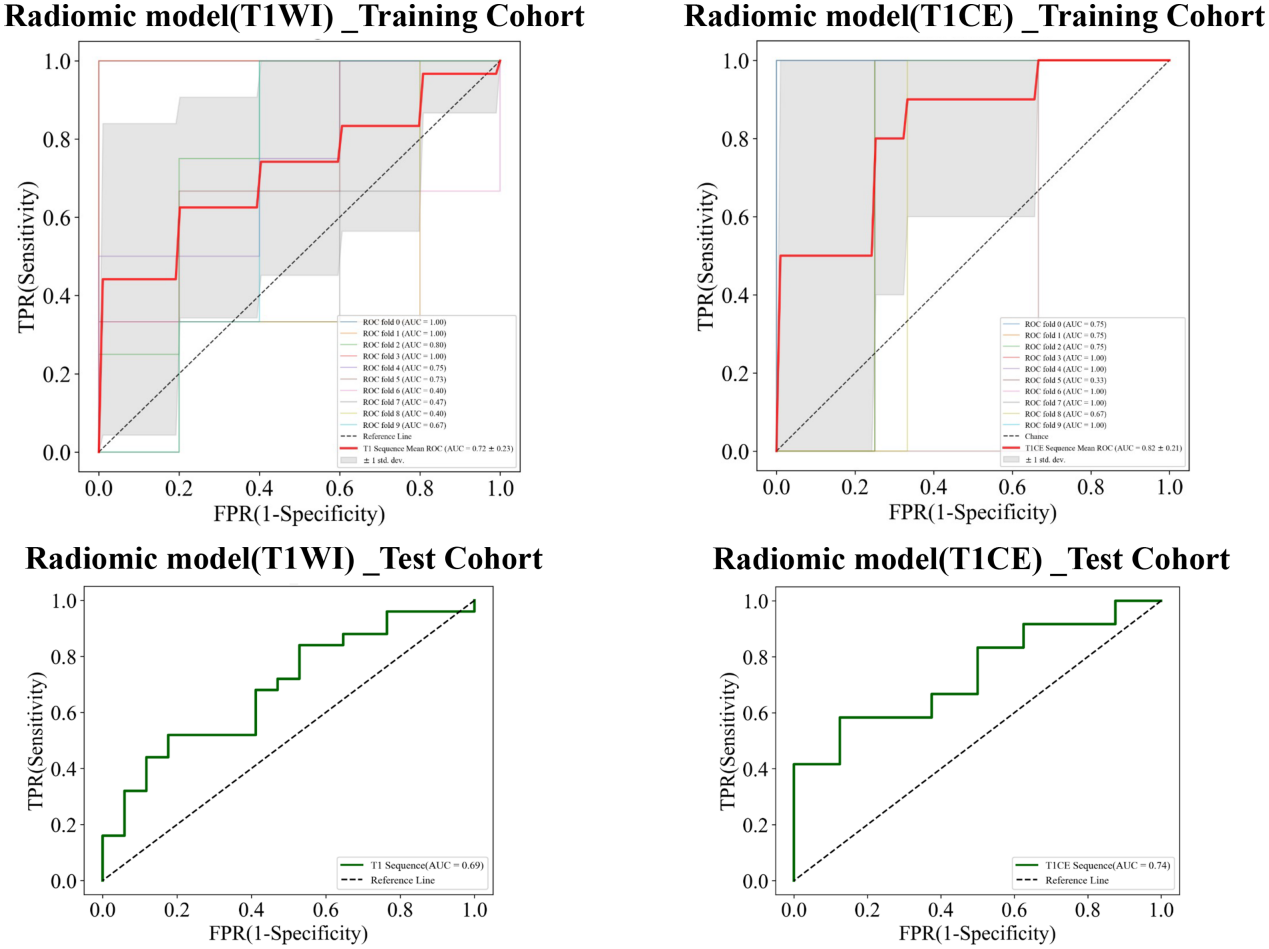
Receiver operating characteristic (ROC) curves of the radiomics_T1WI model and the radiomics_T1CE model in the training and test cohorts, respectively.

The AUC value of the training cohort of the radiomics_T1WI model was 0.72, while the AUC value of the test cohort was 0.69. The AUC value of the training cohort of the radiomics_T1CE model was 0.82, while the AUC value of the test cohort was 0.74 (Figure 7).

### Combined model

**Figure 8:**
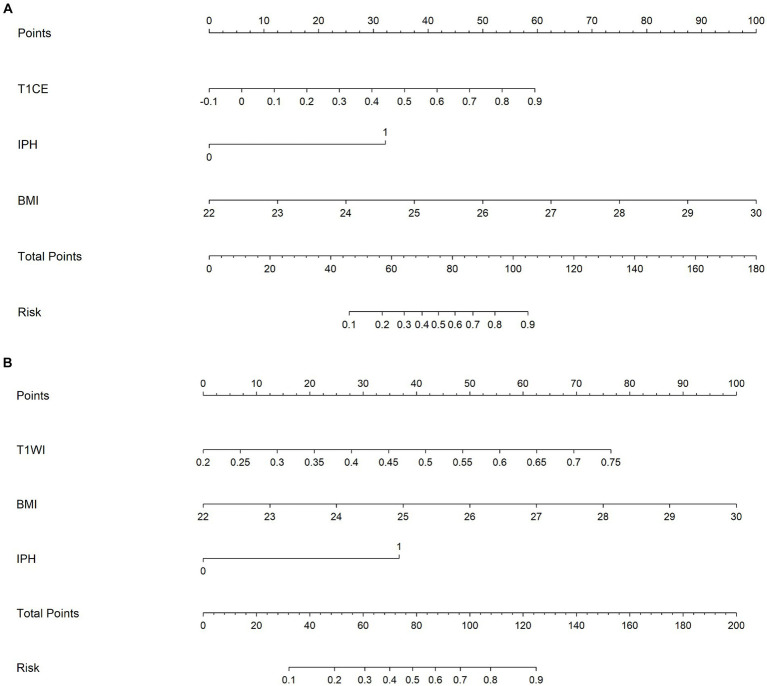
A nomogram which integrates the radiomics scores and traditional features of the training cohort. From the left to right, the probability scoring of ischemic stroke is marked on each axis and increases. An example of how to calculate the total points of a plaque on T1CE and predict the risk of the patient’s ischemic stroke was as follows: a carotid plaque with IPH and the BMI of the patient is 25 receives 32.5 + 37.5 = 70 points from traditional features. A radiomics score of 0.7 corresponds to 47.5. Therefore, this patient scored 117.5 on the nomogram, which indicates a risk of ischemic stroke over 90%. **(A)** Combined_TICE model nomogram. **(B)** Combined_TIWI model nomogram.

**Figure 9:**
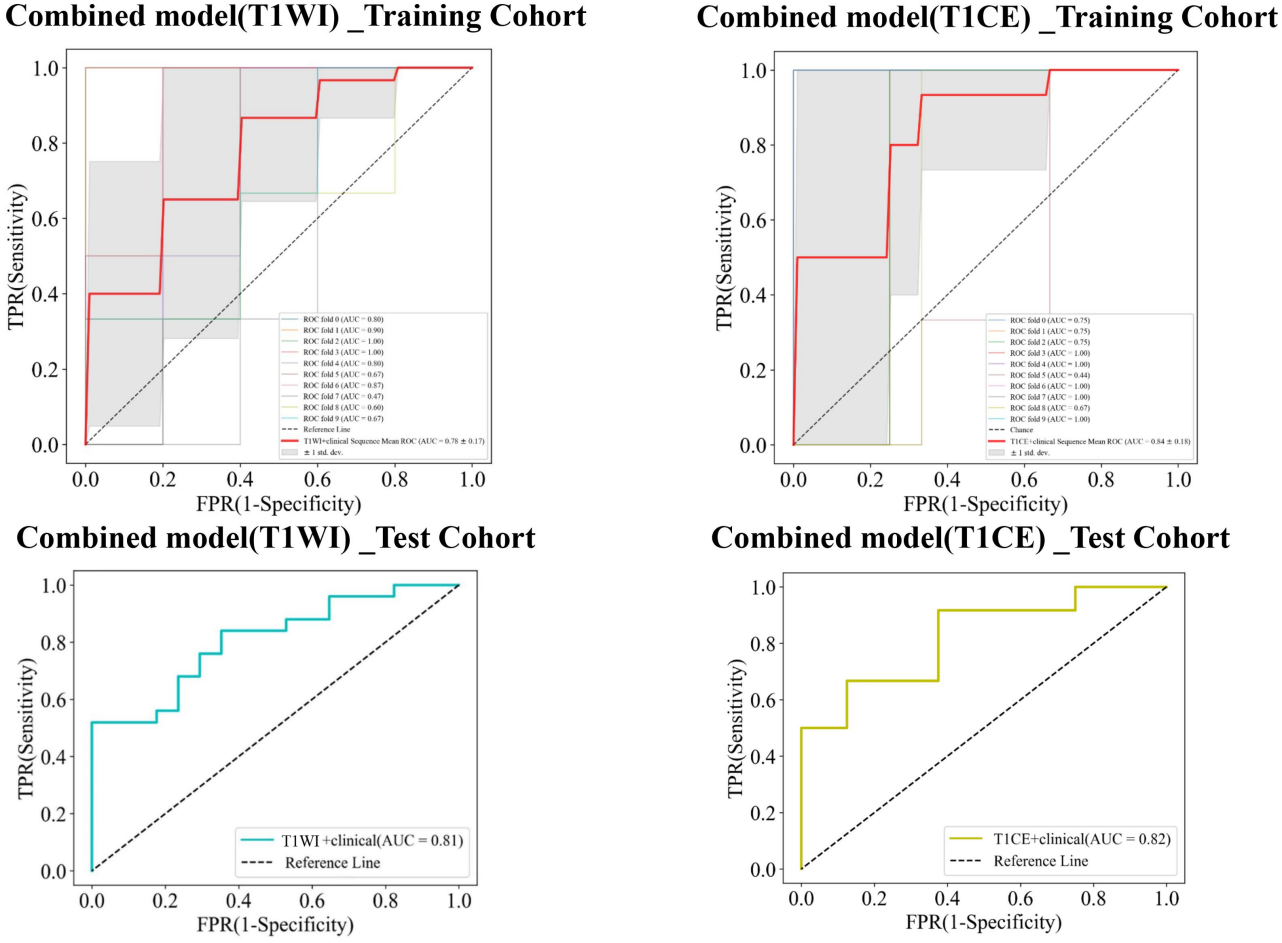
Receiver operating characteristic (ROC) curves of the combined_T1WI model and the combined_T1CE model in the training and test cohorts, respectively.

Finally, combined models were constructed and displayed as nomograms (Figure 8). In the training cohort, the combined_T1WI model exhibited an AUC value of 0.78, which was 0.81 in the test cohort. The combined_T1CE model exhibited an AUC value of 0.84 in the training cohort and 0.82 in the test cohort (Figure 9).

Table 3 lists the specificity, sensitivity, accuracy, AUC, and negative predictive value (NPV) and positive predictive value (PPV) of the traditional, radiomics, and combined models. The combined_T1CE model showed a higher AUC value than the other models.

**Table 3:** Predictive ability of all models.

	Cohort	Sensitivity	Specificity	Accuracy	AUC	NPV	PPV
Traditional model	Training	0.61	0.78	0.70	0.79	0.73	0.69
	Test	0.69	0.85	0.77	0.78	0.70	0.85
Radiomics_T1WI model	Training	0.59	0.82	0.74	0.72	0.79	0.67
	Test	0.32	0.88	0.55	0.69	0.47	0.80
Radiomics_T1CE model	Training	0.72	0.82	0.77	0.82	0.80	0.77
	Test	0.50	0.88	0.65	0.74	0.54	0.86
Combined_T1WI model	Training	0.25	0.80	0.56	0.78	0.57	0.50
	Test	0.52	0.94	0.69	0.81	0.57	0.93
Combined_T1CE model	Training	0.82	0.78	0.79	0.84	0.86	0.79
	Test	0.50	0.88	0.70	0.82	0.57	0.84

## Discussion

At present, the research on the relationship between carotid atherosclerotic plaque and ischemic stroke mainly focuses on the assessment of basic imaging characteristics of plaque or the degree of vascular stenosis. Previous studies have reported that the composition of atherosclerotic plaque is closely related to the occurrence of ischemic stroke (21). However, there are limitations in exploring the relationship between plaques and ischemic stroke based solely on their basic imaging features. First, clinical risk factors such as age, gender, hypertension, hyperglycemia, hyperlipidemia, obesity, smoking, and alcohol consumption are closely related to ischemic stroke. Second, the evaluation of the basic imaging features of plaques is subjective and qualitative, and the results are greatly influenced by the personal factors of the researchers. However, beyond this traditional evaluation method, carotid plaque imaging combined with artificial intelligence to accurately predict the risk of ischemic stroke is needed. Radiomics convert medical images to quantitative indicators through high-throughput extraction by data evaluation algorithms for predicting the risk of disease (22, 23). Therefore, carotid plaque VOIs were delineated on HR-VWMRI; traditional, radiomics, and combined models for predicting ischemic stroke were established.

Multivariate logistic regression analysis between stroke group and stroke-free group results showed that BMI and IPH were independent predictors of ischemic stroke. Then, the BMI and IPH were used to build the traditional model. The results revealed that the AUC of the traditional model is 0.79 in the training cohort and 0.78 in the test cohort. BMI is a commonly used indicator to measure the degree of obesity and thinness of the body in the world, mainly used to reflect the total body fat (24). High BMI is closely related to hypertension, diabetes, and other risk factors, which are collectively called metabolic syndrome (25, 26). Studies found that the components of metabolic syndrome interacted with each other to promote the progress of metabolic disorder *in vivo*, which not only led to intracranial and extracranial atherosclerotic lesions but also led to the impairment of cerebrovascular regulation ability and microcirculation, further promoting the occurrence and development of cerebrovascular diseases dominated by ischemic stroke (27, 28). Atherosclerosis is known to be the main cause of ischemic stroke. Research shows that fat cells in patients with high BMI significantly increase, promote the release of inflammatory cytokines, and change the inflammatory state of the body (29, 30). The formation and development of atherosclerotic plaque is a chronic inflammatory process (31). Therefore, it is believed that high BMI is associated with ischemic stroke (32).

IPH is attributed to fragile neovascularization. The rupture of the neovascular endothelium will increase the stress of the plaque wall, making the plaque wall easier to rupture and cause thrombosis, which is more likely to lead to ischemic stroke (33, 34). Many studies have found that IPH is an independent predictor of stroke events. In patients with symptomatic atherosclerosis, the incidence of IPH is higher than that of asymptomatic patients (35–37). The results of our study revealed that the incidence of IPH in the stroke group was higher than that in the stroke-free group, which was consistent with previous study results. In the training and test cohorts, the AUC value of the radiomics_T1WI model is the lowest, but the prediction performance is significantly improved when the model combines IPH and BMI. In addition, when the radiomics_T1CE model is combined with IPH and BMI, the prediction performance of the model is further improved. This indicates that IPH and BMI are significantly associated with ischemic stroke (38).

The establishment of radiomics_T1WI and radiomics_T1CE models is based on HR-VWMRI 3D T1WI and 3D T1CE images, respectively. The use of HR-VWMRI 3D imaging can better characterize plaque features, including more comprehensive, rich, and detailed image information. 3D imaging methods can reduce local volume effects in 2D imaging and improve the results of radiomics analysis. We found the independent radiomics features from T1WI and T1CE images were different. This is because the signal characteristics in pre- and post-contrast T1WI reflect different pathophysiological characteristics of plaque. For example, the hyperintensity on pre-contrast T1WI is possibly IPH; on the other hand, the hyperintensity on post-contrast T1WI is attributed to the plaque neovascularization or the contrast uptake by active inflammation (39). After applying the LASSO algorithm, 10 features were finally retained based on T1WI including one shape feature, two first-order statistics features, and seven texture features, and eight features were finally retained based on T1CE including two first-order statistics features and six texture features. The first-order statistics features describe the distribution of single voxel value without considering the spatial relationship and are obtained based on histogram analysis and calculation (12). The second-order statistics features are usually described as “texture” features, which describe the statistical relationship between voxels with similar (or different) contrast values (11). In the 18 final features, only first-order statistics features and texture features appeared in the final screening results of the two sequences. It means that these two types of features may be the most important quantitative features to describe plaques, and these features cannot be visually evaluated by radiologists.

Our study revealed that the prediction performance of the radiomics_T1CE model is significantly superior to the radiomics_T1WI model no matter in training or test cohort. This reflects that plaque enhancement is another independent risk factor for ischemic stroke (20, 40). The main reason for plaque enhancement is the increase in neovascularization and endothelial permeability. The contrast agent enters and stays in the plaque through the loose endothelium, resulting in plaque vulnerability and different degrees of enhancement. Therefore, plaque enhancement is closely related to the occurrence of ischemic stroke events (41, 42). However, it is interesting that the results of multivariate logistic regression analysis show that there is no statistically significant difference in plaque enhancement between the stroke group and the stroke-free group. This further shows that radiomics contains more information; for example, enhancement is a sign of high-risk plaques, but most previous studies were subjective visual qualitative recognition, lacking objective quantitative information. However, radiomics can provide quantitative information that is not relevant to the reader, which is difficult to visualize or too numerous for radiologists to visually evaluate (43).

In the training and test cohorts, the combined_T1CE model has the highest AUC value. Compared with the radiomics_T1CE model, the prediction performance of the combined_T1CE model has been improved. However, there is no statistically significant difference between the two models in the training cohort. This could be explained by the relative weights of the radiomics_T1CE model versus the traditional model, and the combined_T1CE model was weighted heavily toward the enhancement of radiomics characteristics, which produced better performances (10).

This study beyond the assessment of the basic imaging characteristics of carotid atherosclerotic plaque or the degree of vascular stenosis uses a new model, that is, the combination of carotid atherosclerotic plaque imaging and artificial intelligence to explore the relationship with ischemic stroke. The study showed that radiomics score, IPH, and BMI were independent indicators of the risk of ischemic stroke. Combined with these independent risk factors, novel radiomics nomograms were generated. The generated nomogram based on T1CE had good predictive value, with AUCs of 0.84 and 0.82 in the training and test cohorts, respectively. These encouraging results deserve further multicenter trials applying carotid plaque radiomics features based on HR-VWMRI T1CE images and clinical characteristics for predicting the risk of ischemic stroke.

Limitations of this study: First, this study was a single center with a relatively small sample size; multicenter and larger data sets are needed to evaluate the prediction performance of the model in future study. Second, VOIs were manually delineated, despite the excellent reproducibility; however, due to the small size of the plaque, manual segmentation is a challenging task that takes a lot of time. Automatic segmentation would improve segmentation efficiency and accuracy. Third, because the boundary of plaque on TOF images is indistinct, and T2WI is not a 3D isovoxel sequence, we did not perform radiomics analysis on it. Fourth, there is a lack of multimodal imaging indicators such as cerebral blood flow and collateral circulation. In future, multimodality imaging combined with artificial intelligence is needed to establish a prediction model of ischemic stroke and improve the primary prevention strategy of stroke.

## Conclusion

As a feasible and exploratory study, this study provides new insights into the prediction of ischemic stroke. The above results indicate that the combined_T1CE model incorporating clinical characteristics and carotid plaque radiomics features based on HR-VWMRI T1CE images can accurately predict the risk of ischemic stroke.

## Data availability statement

The original contributions presented in the study are included in the article/supplementary material, further inquiries can be directed to the corresponding author.

## Ethics statement

The studies involving humans were approved by the Ethics Review Board of Lanzhou University Second Hospital (scientific research project ethics approval number: 2022A-695). The studies were conducted in accordance with the local legislation and institutional requirements. The participants provided their written informed consent to participate in this study.

## Author contributions

NH: Writing – original draft, Methodology. WH: Writing – review & editing. YM: Methodology, Writing – review & editing. YZ: Data curation, Writing – review & editing. SY: Validation, Writing – review & editing. LM: Supervision, Writing – review & editing. JL: Formal analysis, Writing – review & editing. JZ: Funding acquisition, Project administration, Supervision, Writing – review & editing.

## Footnotes

^1^ www.itk-snap.org

^2^ https://www.python.org
